# The influence of demographic and meteorological factors on temporal patterns of rotavirus infection in Dhaka, Bangladesh

**DOI:** 10.1098/rspb.2021.2727

**Published:** 2022-06-08

**Authors:** Ernest O. Asare, Mohammad A. Al-Mamun, Monira Sarmin, A. S. G. Faruque, Tahmeed Ahmed, Virginia E. Pitzer

**Affiliations:** ^1^ Department of Epidemiology of Microbial Diseases, Yale School of Public Health, Yale University, New Haven, CT, USA; ^2^ Department of Pharmaceutical Systems and Policy, School of Pharmacy, West Virginia University, USA; ^3^ Nutrition and Clinical Services Division (NCSD), International Centre for Diarrheal Disease Research, Bangladesh (icddr,b), Dhaka, Bangladesh

**Keywords:** rotavirus seasonality, rotavirus transmission, meteorological factors, birth rate, demography

## Abstract

To quantify the potential impact of rotavirus vaccines and identify strategies to improve vaccine performance in Bangladesh, a better understanding of the drivers of pre-vaccination rotavirus patterns is required. We developed and fitted mathematical models to 23 years (1990–2012) of weekly rotavirus surveillance data from Dhaka with and without incorporating long-term and seasonal variation in the birth rate and meteorological factors. We performed external model validation using data between 2013 and 2019 from the regions of Dhaka and Matlab. The models showed good agreement with the observed age distribution of rotavirus cases and captured the observed shift in seasonal patterns of rotavirus hospitalizations from biannual to annual peaks. The declining long-term trend in the birth rate in Bangladesh was the key driver of the observed shift from biannual to annual winter rotavirus patterns. Meteorological indices were also important: a 1°C, 1% and 1 mm increase in diurnal temperature range, surface water presence and degree of wetness were associated with a 19%, 3.9% and 0.6% increase in the transmission rate, respectively. The model demonstrated reasonable predictions for both Dhaka and Matlab, and can be used to evaluate the impact of rotavirus vaccination in Bangladesh against changing patterns of disease incidence.

## 1. Introduction

Rotavirus remains an important diarrhoeal disease among children less than 5 years of age globally with significant morbidity and mortality in low- and middle-income countries (LMICs), particularly in sub-Saharan Africa and South Asia. Together, these two regions accounted for more than 85% of estimated global rotavirus deaths among children less than 5 years between 2000 and 2013 [1]. However, rotavirus vaccine impact has been found to be low to moderate in these regions [2]. In addition, the cost of treatment for rotavirus episodes imposes an economic burden on households in South Asia [3,4]. In order to quantify vaccine impact and identify strategies to improve both the performance and cost-effectiveness of rotavirus vaccines in these settings, a better understanding of the drivers of pre-vaccination rotavirus patterns is required.

In Bangladesh, rotavirus is the leading cause of severe diarrhoea, accounting for about two-thirds of acute gastroenteritis (AGE) hospitalizations and about 3% of all deaths among children less than 5 years old [5,6]. Rotavirus transmission occurs year-round with a predominant seasonal peak during the dry season (November–February). Almost half of hospitalizations occur among infants 6–11 months old [6,7]. Several studies reported an association of rotaviral diarrhoea with hypernatremia and convulsion in children [8]. The high morbidity but relatively low mortality rates in Bangladesh can be attributed in part to the widespread availability of oral rehydration salts for early treatment at home [9,10]; the future introduction of rotavirus vaccine is likely to further reduce the morbidity and mortality rate.

In Bangladesh, changes in the epidemiology of rotavirus such as increases in the proportion of diarrhoea attributable to rotavirus and genotype circulation have been reported [5,11,12]. In order to evaluate the impact of rotavirus vaccination against changing epidemiological patterns of disease burden, it is necessary to identify potential drivers of rotavirus incidence in the absence of vaccination. Some of these drivers of pre-vaccination rotavirus epidemiology could also interfere with vaccine performance and contribute to the differential rotavirus vaccine impact between and within LMICs and high-income settings [13–16].

A number of studies have attempted to assess the importance of demographic and environmental factors as potential drivers of the spatio-temporal pattern of rotavirus incidence [17–21]. For instance, Pitzer *et al*. [18] demonstrated that high birth rates in tropical countries may contribute to the observed lack of rotavirus seasonality in these settings. Nevertheless, meteorological variables are likely to influence rotavirus seasonal patterns. Temperature and rainfall have been found to be important factors controlling both virus survival outside the host and dispersal and persistence in the environment [22,–24]. In South Asia, Jagai *et al*. [25] found an inverse relationship between both temperature and rainfall and the risk of rotavirus infections, while Hasan *et al*. [26] found a positive association between rotavirus and diurnal temperature variations.

While most modelling studies have assessed the impact of demographic and environmental factors independently, further studies are needed to assess their combined effects on rotavirus incidence. Here, we use mathematical models of rotavirus transmission dynamics to investigate the importance of long-term and seasonal variations in the birth rate and meteorological indices, including diurnal temperature range (*dtr*), degree of wetness (*dow*) and surface water presence (*wpre*), as potential drivers of temporal variations of rotavirus incidence in Dhaka, Bangladesh. We compared 23 years of observed weekly rotavirus cases with those predicted by models with and without incorporating seasonal variation in the birth rate and meteorological indices. For the model to be used to evaluate future benefits of rotavirus vaccination in Bangladesh, it is important that the model can reproduce the observed pre-vaccination rotavirus patterns. Thus, the predictive potential of the models was assessed by performing external validation using weekly data between 2013 and 2019 from Dhaka and Matlab.

## 2. Methods and data

### (a) Rotavirus data

Weekly records of confirmed rotavirus-positive cases for Dhaka were obtained from the International Centre for Diarrheal Disease Research, Bangladesh (icddr,b). The surveillance platform has been described previously [5,27,29]. Briefly, stool samples were collected from a systematic sample of diarrhoeal patients presenting to iccdr,b hospital in Dhaka; every 25th patient (4%) was sampled between 1990 and 1995, while every 50th patient (2%) has been sampled since 1996. Stool samples were tested for rotavirus using ELISA. The rotavirus-positive cases were aggregated into 17 age groups (monthly intervals for infants less than 1-year-old, yearly intervals from 1 year to less than 5 years of age, and those aged 5 years or older). To examine the potential changes in rotavirus patterns over time, we divided the dataset into two parts: 1990–2001 (part 1) and 2003–2012 (part 2). The data for 2002 were used as a transition period to evaluate the best-fit models from the sub-data. In addition, the best-fit models were validated using weekly confirmed rotavirus-positive cases between 2013 and 2019 (part 3).

We also performed external model validation using weekly rotavirus surveillance data obtained from Matlab Hospital between 2013 and 2019. Matlab is a rural area situated 55 km southeast of Dhaka and forms part of the icddr,b's Diarrhoeal Disease Surveillance System (DDSS). Unlike Dhaka, stool samples are collected from all diarrhoeal patients coming from the DDSS area and attending the Matlab Hospital and tested for rotavirus using ELISA. Similar to Dhaka, the rotavirus-positive cases were aggregated into 17 age groups.

### (b) Meteorological data and indices

Daily minimum and maximum 2 m air temperature data were obtained from the European Centre for Medium-Range Weather Forecasts (ECMWF) ERA-Interim reanalysis product [30], while daily rainfall data were obtained from Climate Hazards group Infrared Precipitation with Stations (CHIRPS) [31]. These are reanalysed data that combine model outputs with earth observational datasets. Previous studies have used hydrometeorological variables derived from these earth observations to predict rotavirus [32,33]. Both datasets were extracted over Dhaka and Matlab for the study period 1990–2019. We used these meteorological variables to derive three indices: diurnal temperature range (*dtr*), degree of wetness (*dow*) and surface water presence (*wpre*).

The daily *dtr* is given by

2.1 $$dtr=T_{\max}-T_{\min},$$

where *T*_max_ and *T*_min_ are the daily maximum and minimum temperatures, respectively. The daily *dtr* was normalized and then averaged to generate a weekly time series. We used *dtr* rather than temperature because it better captures the combined effects of cooler night-time temperatures and lower humidity, both of which favour virus survival [34]; furthermore, it demonstrated strong associations with rotavirus incidence in Dhaka in a previous analysis [26].

The weekly *dow* [35] is given by

2.2 $$\begin{array}{rl} & dow\\ & =\frac{\left(\mathrm{total}\;\mathrm{rainfall}\;\mathrm{per}\;\mathrm{week})\times (\mathrm{number}\;\mathrm{of}\;\mathrm{rainy}\;\mathrm{days}\;\mathrm{per}\;\mathrm{week}\right)}{7}. \end{array}$$

The *dow* was normalized before incorporating it into the model. We used the *dow* instead of actual rainfall to reduce sensitivity to isolated heavy storm events within a week.

The daily *wpre* is an output from a simple water balance model developed by Asare *et al*. [36,37] that requires only rainfall data and other estimated fluxes to predict fractional coverage of surface water over a given area. The model equation is given by

2.3 $$\begin{array}{rl} \frac{dw_{\mathrm{pond}}}{dt} & =\frac{2}{\rho h_{\mathrm{ref}}}{\left(\frac{w_{\mathrm{ref}}}{w_{\mathrm{pond}}}\right)}^{\rho /2}((Q(w_{\max}-w_{\mathrm{pond}})\\ & \;+\;Pw_{\mathrm{pond}})(1-f)-w_{\mathrm{pond}}(E+fI_{\max})), \end{array}$$

where *w*_pond_ is the daily fractional flood water coverage, *ρ* is the geometrical shape factor, *w*_max_ is the maximum flood water coverage, *h*_ref_ is the reference flood water depth, *w*_ref_ is the reference flood water coverage, *Q* (derived from the soil conservation service curve number method [38]) is the runoff, *P* is the rainfall, *E* is the evaporation, *I*_max_ is the maximum infiltration and $f=w_{\mathrm{pond}}/w_{\max}$. The values of the fixed model parameters are listed in the electronic supplementary material, table S1. The daily fractional flood water coverage was normalized and aggregated to generate a weekly time series. The *wpre* provides a proxy for flooding events.

Since *dow* and *wpre* are correlated and highest during the monsoon season, we evaluated them in separate models with *dtr*, which peaks in the winter season. To interpret the effect sizes associated with each meteorological variable, we multiplied by the standard deviation to convert back to the original scale.

### (c) Demographic data

Data on long-term trends in the crude birth rate, crude death rate, average annual rate of population change and total population for Dhaka were obtained from the World Urbanization Prospects database [39]. We used linear interpolation to estimate values for the weekly birth and death rates per 1000 population, and estimated the net rate of immigration as the average annual rate of population change minus the difference between the crude annual birth and death rates. We verified that our model was able to reproduce the observed total size and age distribution of the Dhaka population over time.

To explore the potential impact of seasonality in the birth rate, we used data from a longitudinal study of about 2500 women across 14 villages in Matlab, Bangladesh that were followed for a period of 4 years [40]. As the birth seasonality in Dhaka is unknown, we assumed that it was either strongly seasonal as observed in Matlab or exhibited no seasonality. We fitted a sinusoidal function to the monthly birth rate data from Matlab to estimate the amplitude and timing (i.e. phase) of seasonality in the birth rate, then multiplied the resulting function by the birth rate of Dhaka to obtain the seasonal birth rate (equation 2.4) used in the models:

2.4 $$B_{s}(t)=B_{lt}(t)\times \left(1+0.42\times \cos\left(\frac{2\pi t-48}{52.18}\right)\right),$$

where *B_s_* and *B_lt_* are the seasonal and long-term trend in birth rates for Dhaka, respectively. We assumed that the amplitude and timing of birth seasonality remained consistent over time.

### (d) Model description

**Figure 1.**
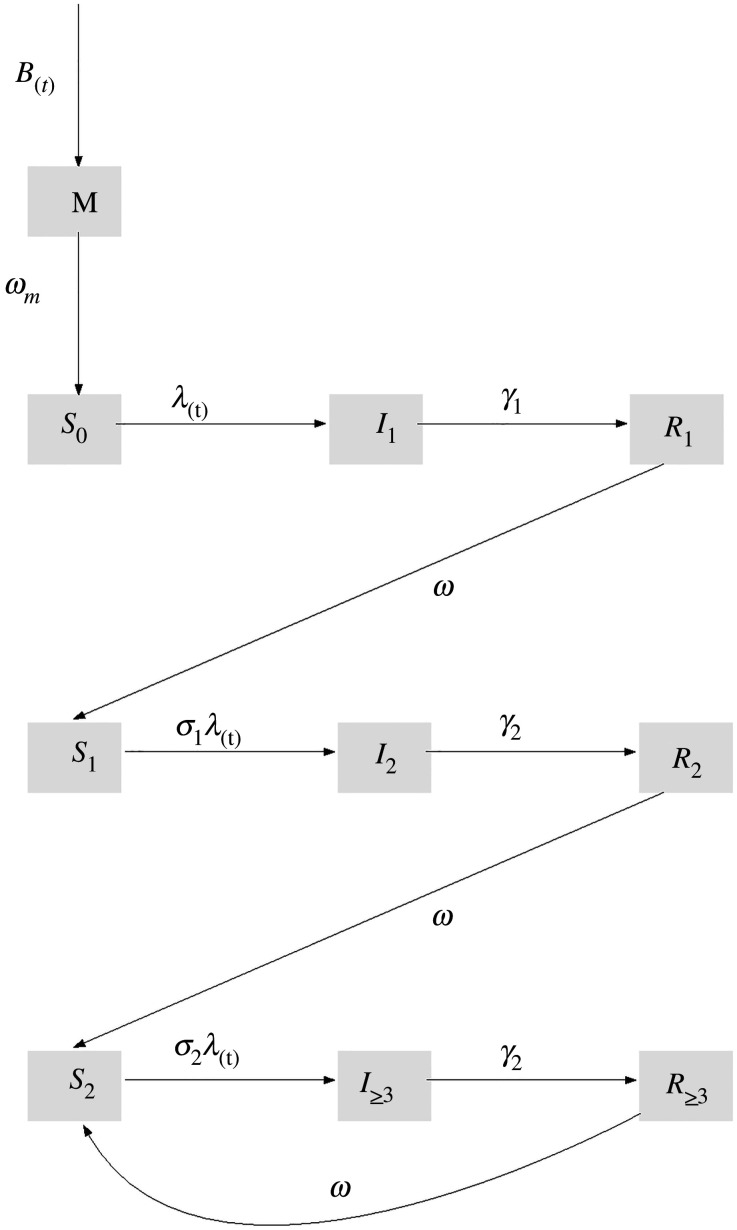
Schematic of the mathematic model for rotavirus transmission dynamics. Model parameters are described in electronic supplementary material, table S2.

We modified the mathematical model of rotavirus transmission developed by Pitzer *et al*. [17] (figure 1) to incorporate seasonal variation in the birth rate and meteorological indices. The model assumes that newborns enter the maternal compartment *M* (which was divided into six sub-compartments to account for non-exponential waning of maternal antibodies) at a rate equal to the birth rate *B* and are protected from rotavirus infection by maternal antibodies. This immunity wanes at a rate *ω_m_*, after which infants become fully susceptible to primary infection (*S_0_*). Primary infections occur at a rate *λ*, and infected individuals (*I_1_*) remain infectious for an average period of 1*/γ_1_*, with a fraction (*d_1_*) developing severe rotavirus diarrhoea. Recovered individuals enter the *R*_1_ compartment and are assumed to have temporary immunity to reinfection that wanes at a rate *ω*. Following the waning of immunity, individuals become susceptible to secondary infection (*S_1_*), which occur at a reduced rate *σ*_1_*λ*. The secondary infected individuals (*I*_2_) are less likely to develop severe diarrhoea (*d*_2_), have lower infectiousness (by a factor *ρ_2_*) and recover at a faster rate *γ_2_* into the *R_2_* compartment. Again, immunity wanes at the same rate *ω*, after which individuals move into the partially immune susceptible compartment (*S_2_*); subsequent infections occur at a further reduced rate *σ_2_λ*, but are assumed to be mostly asymptomatic or mildly symptomatic (proportion severe *d*_3_ was estimated). The partially immune infected individuals (*I*_≥3_) have infectiousness reduced by a factor *ρ*_≥3_ and recover at a rate *γ_2_*. The recovered individuals enter the *R*_≥3_ compartment, where their immunity wanes at the same rate *ω*, after which individuals return to the *S_2_* compartment.

The force of infection (i.e. the rate of transmission from infected to susceptible individuals) at time *t* in weeks, *λ*(*t*), is given by

2.5 $$\lambda (t)=\beta (t)(I_{1}(t)+\rho_{2}I_{2}(t)+\rho_{\geq 3}I_{\geq 3}(t)),$$

where *β*(*t*) is the seasonally varying transmission parameter. To determine the number of harmonic terms to include in the model, we performed wavelet analysis on the weekly rotavirus-positive time-series data for 1990–2019 and subsets of data divided into part 1 (1990–2001), part 2 (2003–2012) and part 3 (2013–2019; validation period).

For our default model (model 0), we assumed sinusoidal forcing with both annual and biannual harmonic terms (equation (2.6*a*)):

Model 0:

2.6*a* $$\beta (t)=\beta_{0}\left(1+b_{1}\cos\left(\frac{2\pi t-\emptyset_{1}}{52.18}\right)+b_{2}\cos\left(\frac{2\pi t-\emptyset_{2}}{26.09}\right)\right).$$

We then extended this model to estimate the contribution of meteorological forcing in addition to the sinusoidal forcing, evaluating *dtr* and either *dow* (model A, equation (2.6*b*)) or *wpre* (model B, equation (2.6*c*)) as best explaining the influence of rainfall:

Model A:

2.6*b* $$\begin{array}{rl} \beta (t) & =\beta_{0}(1+b_{1}\cos\left(\frac{2\pi t-\emptyset_{1}}{52.18}\right)+b_{2}\cos\left(\frac{2\pi t-\emptyset_{2}}{26.09}\right)\\ & \;+\;b_{dtr}(dtr)+b_{dow}(dow)), \end{array}$$

Model B:

2.6*c* $$\begin{array}{rl} \beta (t) & =\beta_{0}(1+b_{1}\cos\left(\frac{2\pi t-\emptyset_{1}}{52.18}\right)+b_{2}\cos\left(\frac{2\pi t-\emptyset_{2}}{26.09}\right)\\ & \;+\;b_{dtr}(dtr)+b_{wpr}(wpre)), \end{array}$$

where *β_0_* is the baseline transmission rate, *b*_1_ is the amplitude of annual seasonal forcing, *b*_2_ is the amplitude of biannual seasonal forcing, *ϕ*_1_ is the annual seasonal offset, *ϕ*_2_ is the biannual seasonal offset, *b*_*dtr*_ is the scaling parameter for *dtr*, *b*_*dow*_ is the scaling parameter for *dow* and *b*_*wpre*_ is the scaling parameter for *wpre*. The model input parameters are defined in the electronic supplementary material, table S2.

### (e) Model-fitting approach

We fitted the models to the 23 years of weekly age-stratified rotavirus surveillance data from Dhaka Hospital using a maximum-likelihood framework; we estimated 8 to 10 parameters while fixing the remaining model parameters at values identified from the literature (see electronic supplementary material, table S2). We assumed that the confirmed cases of rotavirus in each week and age group followed a Poisson distribution with mean equal to the model-predicted number of severe rotavirus cases multiplied by an estimated reporting fraction (*h*). The 95% confidence intervals for parameters of interest (*R*_0_, 1/*ω_m_*, *b*_1_, *ϕ*_1_, *b*_2_, *ϕ*_2_, *b*_*dtr*_, *b*_*dow*_ and *b*_*wpre*_) were determined by profile likelihoods. We fitted the models with and without incorporating seasonal variation in the birth rate. The models were fitted to both the whole time series (complete: 1990–2012) and subsets of the data (part 1: 1990–2001 and part 2: 2003–2012) to estimate the model parameters. Finally, we performed external model validation by comparing model predictions to weekly rotavirus surveillance data between 2013 and 2019 from Dhaka and Matlab. We used demographic data for Matlab obtained from the World Urbanization Prospects database [39].

## 3. Results

**Figure 2.**
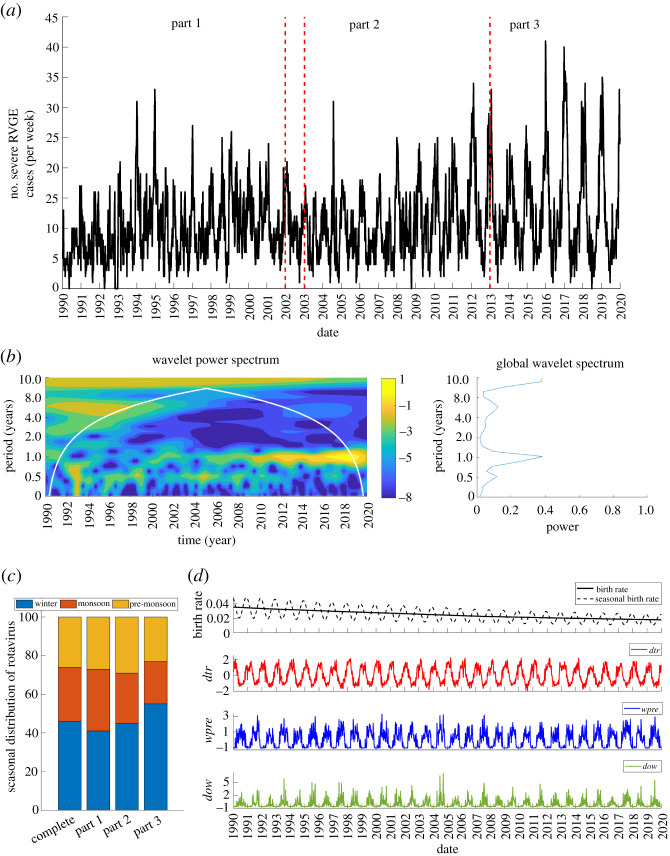
Time series of observed rotavirus cases and demographic and meteorological model inputs. (*a*) Weekly rotavirus cases in Dhaka between 1990 and 2019. The vertical lines indicate the three time periods (part 1: 1990–2001; part 2: 2003–2012 and part 3: 2013–2019). (*b*) The local and global wavelet spectrum of the weekly rotavirus cases. For the local wavelet power spectrum, blue corresponds to lowest intensity and yellow to the highest intensity. (*c*) Stacked bar plot showing the seasonal distribution of rotavirus cases for the complete and sub-divided datasets. (*d*) Weekly interpolated birth rate, corresponding seasonality in the birth rate, and normalized meteorological indices used in the model: diurnal temperature range (*dtr*), surface water presence (*wpre*) and degree of wetness (*dow*).

The average annual incidence of rotavirus was more than 500 confirmed cases in Dhaka between 1990 and 2019. The highest number of confirmed rotavirus cases per week was 41 (figure 2*a*). Observed rotavirus cases showed a shift in seasonal patterns from predominantly biannual to annual peaks towards the end of the model-fitting period (1990–2012) (figure 2*b*). The annual epidemics persisted and increased in intensity during 2013–2019 (validation period) (figure 2*b*). Rotavirus cases occurred year-round, with the highest proportion of cases (46%) occurring during the winter months (November–February) and the peak occurring either in December or January. Minor peaks also usually occurred during the monsoon season (July–October), but occasionally during the pre-monsoon hot season (March–June), accounting for about 27% and 26% of the cases, respectively (figure 2*c*).

Comparing part 1 (1990–2001) and part 2 (2003–2012) (figure 2*c*), there was a slight increase in the proportion of cases occurring in the winter months (part 1: 41%; part 2: 45%) and a reduction in the proportion of cases occurring during the monsoon months (part 1: 32%; part 2: 26%). While there were more cases during the monsoon season than the pre-monsoon season in part 1, the proportion of cases in these two seasons were similar in part 2. Peak cases occurred in either December or January for part 1, but predominantly in January for part 2. For part 3 (validation period), more than half (55%) of rotavirus cases occurred during the winter months (figure 2*c*). There was a comparable proportion of cases occurring during the remaining periods (monsoon: 22%; pre-monsoon: 23%).

Figure 2*d* shows the temporal patterns of the demographic and meteorological drivers used for the model. The birth rate showed a consistent substantial decrease from 36 births per 1000 people in 1990 to 18 births per 1000 people in 2019. Diurnal temperature range peaked during winter, while both surface water presence and degree of wetness peaked during the monsoon periods (figure 2*d*).

**Figure 3.**
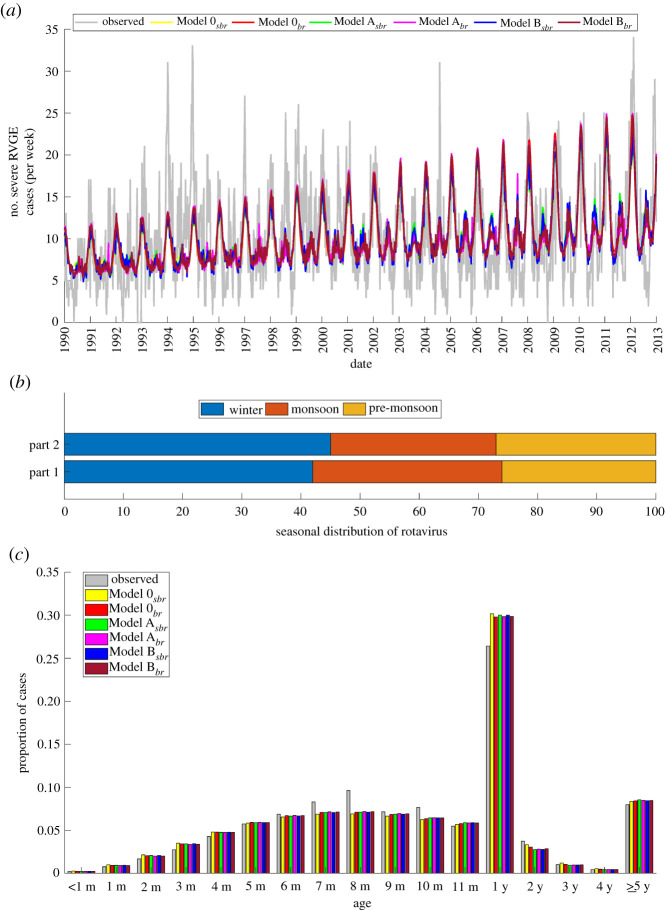
Comparison of model-simulated and observed rotavirus incidence in Dhaka for the complete dataset. (*a*) The weekly time series are plotted; the coloured lines correspond to the best-fit models, while the grey line corresponds to the observed data. (*b*) Comparison of the seasonal distribution of model-simulated rotavirus cases in part 1 and part 2. (*c*) Age distribution of rotavirus cases for the observed data (grey bars) and best-fit models (coloured bars).

All the models were able to reproduce the overall temporal patterns in rotavirus incidence, particularly the shift from biannual to annual seasonal peaks and the increasing trend in the peak season outbreaks of rotavirus towards the end of the study period (figure 3*a*). The models also performed well in estimating the proportion of rotavirus cases occurring in the winter and monsoon seasons (figures 2*c* and 3*b*). Table 1 summarizes the estimated parameter sets that provided the best fit to the complete data and the associated Bayesian information criterion (BIC) values. Based on the BIC, models incorporating both the seasonal birth rate, *dtr* and *wpre* meteorological indices (model *B*_*sbr*_) provided the best fit to the data (table 1). However, the models mostly underestimated the winter peaks in the early part of the study period, specifically 1994–1995 and 1997, and also failed to capture a notable monsoon outbreak in 2004 (figure 3*a*).

**Table 1.** The best-fit model parameters for the complete dataset (1990–2012). Values in parentheses indicate 95% confidence intervals. Here, sbr represents the models using seasonal birth rate, while br represents models assuming a non-seasonal birth rate. The best model (model *B*_*sbr*_) is italicized. *R*_0_: basic reproductive number, *ω_m_*: duration of waning maternal immunity, *d*_3_: proportion of subsequent infections that are severe, *h*: proportion of severe diarrhoea cases reported, *b*_1_: amplitude of annual seasonal forcing, *ϕ*_1_: annual seasonal offset, *b*_2_: amplitude of biannual seasonal forcing, *ϕ*_2_: biannual seasonal offset, *b_dtr_*: scaling parameter for *dtr*, *b_dow_*: scaling parameter for *dow*, *b_wpre_*: scaling parameter for *wpre* and BIC: Bayesian information criterion.

parameter	model 0		model A		model B	
	sbr	br	sbr	br	*sbr*	br
*R*_0_	49 (48.6–49.5)	51.9 (51.1–52.8)	26.7 (26.5–27.0)	35.7 (35.2–36.4)	*26.2* (*25.9–26.4*)	39.3 (38.8–40.1)
1/*ω_m_* (weeks)	26.9 (26.5–27.2)	28.1 (27.6–28.5)	28.9 (28.5–29.2)	29.1 (28.6–29.5)	*28.7 (28.6–29.1)*	28.9 (28.4–29.3)
*d*_3_	4.10 × 10^−4^	3.90 × 10^−4^	3.60 × 10^−4^	3.70 × 10^−4^	*3.60 × 10^−4^*	3.70 × 10^−4^
*h*	0.043	0.042	0.051	0.045	*0.051*	0.045
*b*_1_	0.116 (0.115–0.118)	0.095 (0.088–0.101)	0.103 (0.098–0.126)	0.106 (0.092–0.116)	*0.114 (0.107–0.130)*	0.095 (0.086–0.106)
*ϕ*_1_ (weeks)	10.8 (10.6–11.0)	12.6 (12.1–13.0)	4.761 (4.7–7.0)	11.8 (11.0–12.5)	*5.7 (5.4–8.6)*	11.9 (11.2–12.6)
*b*_2_	0.022 (0.022–0.024)	0.023 (0.022–0.025)	0.046 (0.044–0.051)	0.036 (0.033–0.038)	*0.047 (0.045–0.050)*	0.032 (0.029–0.034)
*ϕ*_2_ (weeks)	33.2 (33.0–33.5)	33.5 (33.2–33.8)	32.6 (32.2–32.8)	33.2 (32.8–33.5)	*32.5 (32.3–32.8)*	33.1 (32.75–33.4)
*b*_dtr_			0.357 (0.354–0.364)	0.179 (0.172–0.187)	*0.367 (0.361–0.372)*	0.128 (0.122–0.136)
*b*_dow_			0.148 (0.057–0.293)	0.843 (0.607–0.951)		
*b*_wpre_					*0.244 (0.175–0.481)*	0.385 (0.266–0.495)
BIC	34 111	34 158	34 065	34 126	*34 036*	34 114

The proportion of rotavirus cases in the monthly less than 1-year-old age groups increased steadily until 8 months of age (figure 3*c*). Over half of the cases (51%) occurred in infants aged 5–11 months, while only 3% of cases occurred in infants less than 3 months of age. Only 5% of cases occurred in 2- to 4-year-olds, and 8% of cases were in persons aged greater than or equal to 5 years (figure 3*c*). Generally, the models were able to reproduce the observed age distribution patterns and accurately predicted both the trend and peak age of hospitalizations (8 months) for infants less than 1 year old (figure 3*c*). In addition, the models showed good agreement with the observed trend and proportion of cases in children aged 2–4 years and greater than or equal to 5 years (figure 3*c*). However, all models slightly overestimated the proportion of rotavirus cases in the 1-year-old age group and underestimated cases in the 7-, 8- and 10-month-old age groups.

**Figure 4.**
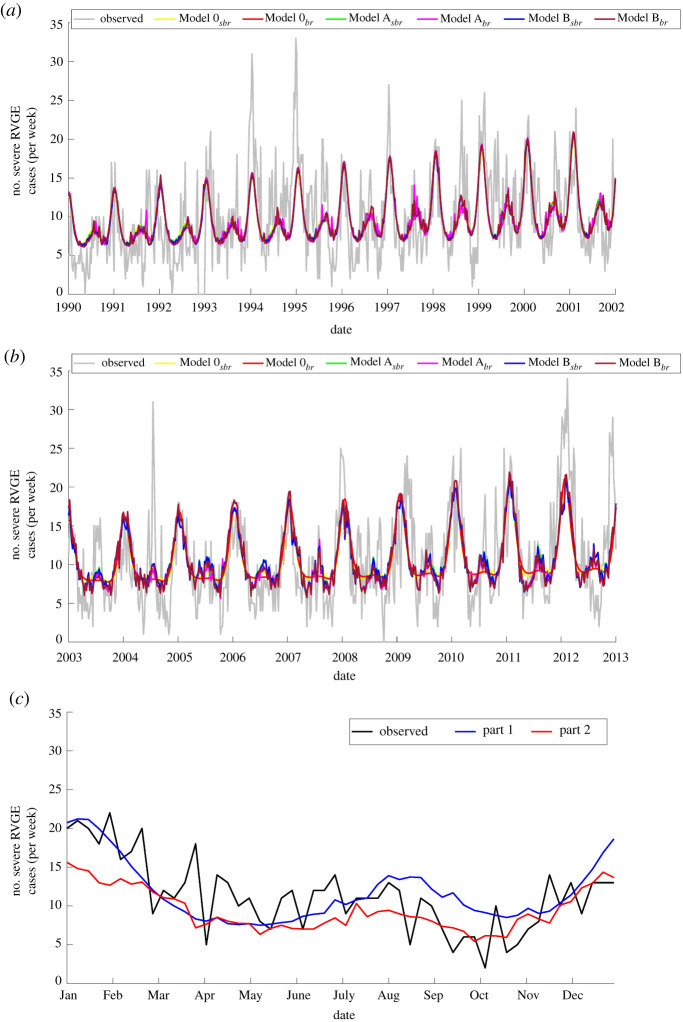
Comparison of models fitted to observed weekly rotavirus cases for during 1990–2001 and 2003–2012 and out-of-sample model validation for 2002. The observed (grey) and model-fitted (coloured lines) number of weekly rotavirus cases was plotted for (*a*) part 1 (1990–2001) and (*b*) part 2 (2003–2012) time series. (*c*) Observed and predicted weekly rotavirus cases in 2002 for the best-fit models (for part 1 and part 2). The comparison of the observed and model-fitted age distributions for part 1 and part 2 are in the electronic supplementary material, figure S1.

Comparing part 1 and part 2, our models were able to estimate the timing of winter peaks and, in addition, the biannual and mostly annual outbreaks in part 1 and part 2 (figure 4*a*,*b*), respectively. The age distribution of rotavirus cases was similar in part 1 and part 2, which was consistent with the estimates from our models (electronic supplementary material, figure S1). Based on BIC, the best model for both part 1 and part 2 is the same model including a seasonal birth rate, *dtr* and *wpre* (electronic supplementary material, table S3). Both best-fit models were able to predict weekly rotavirus patterns in 2002 (figure 4*c*). The best model for part 1 showed a remarkable agreement with the timing and intensity of the winter peak in 2002, but slightly overestimated the monsoon season rotavirus activity. On the other hand, the best-fit model for part 2 satisfactorily predicted monsoon rotavirus activity but underestimated the size of the winter peak.

The relative effect of the meteorological indices on the transmission rate, given by the scaling parameters (*b*_dtr_, *b*_dow_, *b*_wpre_), was greatest for *dtr* (electronic supplementary material, table S4). A 1°C increase in *dtr* was associated with a 19% increase in the rotavirus transmission rate in the best-fit model; this effect was greater for part 2 (34%) compared to part 1 (2.3%). The *wpre* consistently contributed to a greater proportion of the transmission rate (3.9% for a 1% increase in *wpre*) compared to *dow* (0.6% for a 1 mm increase in *dow* for the model including seasonality in the birth rate). The effect estimates for *wpre* and *dow* were generally consistent between part 1 and part 2 (electronic supplementary material, table S4).

**Figure 5.**
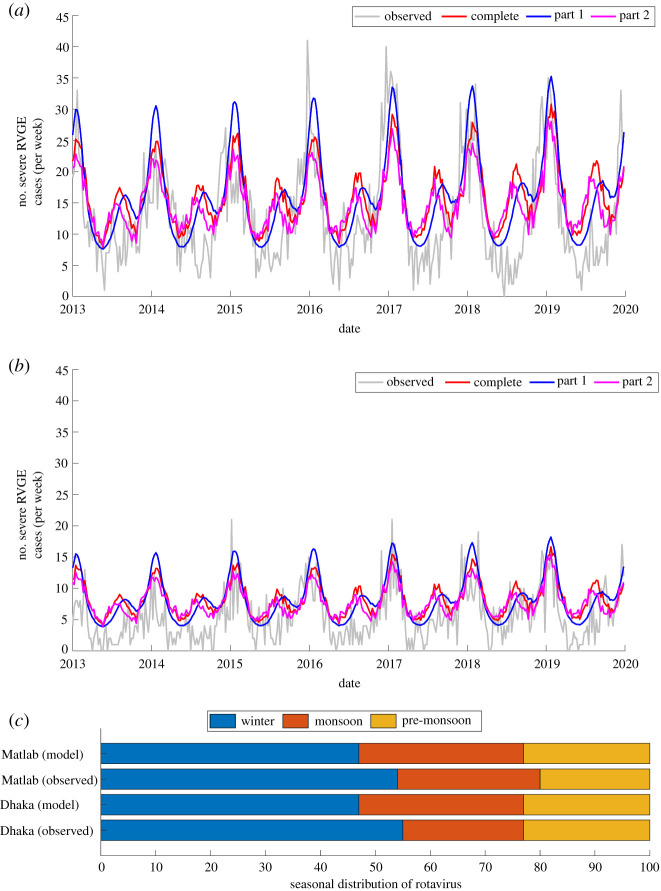
Comparison of model-predicted and observed weekly rotavirus time-series for external model validation. (*a*) Out-of-sample model validation using data from Dhaka. (*b*) External model validation using data from Matlab. (*c*) Comparison of the best-fit model-simulated and observed seasonal distribution in rotavirus cases for both Dhaka and Matlab. In both instances, the best-fit model (model *B*_*sbr*_) was used. (Online version in colour.)

The model validation showed that the best-fit model was able to accurately predict the observed peak timing, duration and intensity of rotavirus activity in winter for both Dhaka and Matlab (figure 5*a*,*b*). However, the best-fit model consistently overestimated rotavirus cases during the pre-monsoon and monsoon seasons. Based on mean absolute error, the best-fit model for part 2 has a slightly better predictive performance for both Dhaka and Matlab (Dhaka complete: 5.89, part 1: 5.33, part 2: 5.30; and Matlab complete: 3.68, part 1: 3.44, part 2: 3.40). The proportion of cases occurring during winter were comparable (Dhaka: 55%; Matlab: 54%), but the proportion of cases occurring in the monsoon season were higher in Matlab than Dhaka (figure 5*c*). The model underestimated and overestimated the proportion of cases occurring during the winter and monsoon seasons, respectively. The models were able to predict the observed trends in the age distribution but over- or underestimated the proportion of cases in some age groups (electronic supplementary material, figure S2).

The intensity of the biannual peak quantified by the wavelet power spectrum was greater than the annual in part 1 (biannual: 0.13; annual: 0.06), but the opposite was true for part 2 (biannual: 0.17; annual: 0.30) and part 3 (biannual: 0.03; annual: 0.85) (electronic supplementary material, figures S3a and S4a). Wavelet analysis of best-fit models with (electronic supplementary material, figure S3b) and without (electronic supplementary material, figure S3c) seasonality in the birth rate shows that both models were able to reproduce the observed shift from the predominantly biannual cycle in part 1 to a stronger annual signal in part 2. However, when we fitted the model assuming a fixed birth rate (using the average birth rate between 1990 and 2012) rather than the declining long-term trend in the birth rate, the model failed to reproduce the observed shift in rotavirus patterns (electronic supplementary material, figure S3d).

There is a substantial increase in the intensity of the annual signal in the validation period (2013–2019) compared to the fitting period for Dhaka (electronic supplementary material, figure S4a). A similar predominant annual signal was observed for Matlab (electronic supplementary material, figure S4b). The best-fit model (model *B*_*sbr*_) was able to predict the observed increase in annual seasonal epidemics of rotavirus (electronic supplementary material, figure S4c,d).

## 4. Discussion

Using mathematical models fitted to weekly rotavirus cases in Dhaka, we demonstrated for the first time that a consistent reduction in the birth rate over time in Bangladesh can help to explain the shift from biannual to annual seasonal patterns. Furthermore, seasonality in the birth rate and meteorological indices are important factors in capturing the amplitude and timing of seasonal peaks, particularly during the winter season. Overall the models demonstrated good performance in predicting both temporal patterns and the age distribution of rotavirus cases in Dhaka and Matlab.

The year-round circulation of rotavirus with major peaks during the winter season (November–February) in Dhaka is similar to what has been found in other parts of the country. Satter *et al*. [6] found that rotavirus contributes to more than 80% of acute AGE hospitalizations among children less than 5 years old between November and February across seven hospitals in Bangladesh. This also is consistent with what has been observed in other tropical regions [25,41].

In addition, we found a shift from biannual to nearly annual rotavirus incidence in Dhaka beginning around 2010, which our models were able to reproduce. Among the demographic and environmental variables considered, the declining long-term trend in the birth rate was identified as a key driver of this shift towards more annual seasonal epidemics. When we fitted the best model (model *B*_*sbr*_) assuming a constant birth rate (using the average birth rate between 1990 and 2012), we could not reproduce the shift from biannual to annual epidemics (electronic supplementary material, figures S3d and S5). This agrees with the study of Park *et al*. [20], who found a high birth rate as an important factor contributing to biannual peaks of rotavirus in Niger. The declining birth rate and an improvement in water treatment have also been hypothesized as potential drivers of a shift towards more seasonal rotavirus epidemics in Spain [42]. Similarly, Pitzer *et al*. [18] demonstrated that countries with lower birth rates tended to experience stronger seasonal rotavirus patterns compared to countries with higher birth rates. There was no significant difference in the meteorological indices between part 1 and part 2 (electronic supplementary material, figure S6), although we found that *dtr* may be contributing more to seasonality in rotavirus transmission during the latter part of the study period. For instance, while there was a more than 14-fold difference in the relative effect of *dtr* on the transmission rate between part 1 and part 2, comparable effects were found for *wpre* and *dow* for the two parts. Given that *dtr* is a useful indicator of climate change, its potential effects on rotavirus incidence under climate change, combined with changes in demographic characteristics, require further detailed investigation. Nevertheless, it is possible that factors such as improvements in sanitation and nutrition [11,43] and changes in rotavirus genotype circulation [44] may play a role in the altered transmission patterns. Future studies should explore whether these factors also contribute to the observed changes in rotavirus patterns.

Comparing models with and without seasonality in the birth rate revealed that the inclusion of seasonal births substantially improved the fit of the models based on BIC scores (table 1; electronic supplementary material, table S3). Although we only had data on birth seasonality in Matlab from the late 1970s, it is possible that seasonality in the birth rate in Bangladesh is substantial. The timing of the birth peak in November (electronic supplementary material, figure S7) may increase the availability of susceptible infants in the winter season, after maternal antibodies have waned, thereby facilitating the winter outbreaks. Since a high proportion of susceptible infants are infected during the winter period, there will be fewer susceptible infants remaining after the winter peak season, particularly during the latter part of the study period (2003–2012) and during the model validation period (2013–2019) when birth rates were lower, leading to decreasing monsoon peaks. Other studies have also demonstrated the importance of birth seasonality in predicting the timing and amplitude of measles epidemics [45,46].

The meteorological indices may also play an important role in predicting the timing of rotavirus epidemics, which predominately occur in January in Dhaka. Thus, meteorological indices derived from earth observations can be incorporated into dynamical models to improve predictions of rotavirus infections. Rotavirus survival is enhanced during cold dry periods, which is supported by the large coefficient of *dtr* estimated for part 2 and the consistency in the peak in *dtr* and rotavirus incidence (electronic supplementary material, figure S6). Other studies have shown that rotavirus peaks in the dry cold period in the tropics and exhibits inverse associations with rainfall, temperature and relative humidity [25,41]. However, variation in the susceptible population likely influences the disease seasonality. This is consistent with findings by Martinez *et al*. [19] who, using the same flood forcing, found differences in population density as a key driver of the observed differences in rotavirus seasonality between the main Dhaka city and peripheral towns surrounding it.

Contrary to the shift in temporal patterns of rotavirus from the 1990s to the 2000s, there was no significant change in the age distribution. The stable age distribution provides the basis for future examination of how vaccination could shift the age distribution of rotavirus cases. There is an agreement between the observed (60%) and predicted (56–58%) proportion of cases among children aged less than 12 months, which is similar to that reported for other LMICs (56–69%) [47–50], but higher than the range reported in high-income countries (41–55%) [51,52] prior to vaccine introduction. The small proportion of cases in infants less than 3 months (approx. 3%) agrees with the 3% observed globally [50] and suggests that the vast majority of rotavirus cases could be prevented using an infant vaccine schedule. Approximately 8% of cases occurred in the greater than or equal to 5 age group (figure 3*c*; electronic supplementary material, figure S1), which is similar to what has been reported in other parts of Bangladesh as well as in other countries [53–55]. This clearly shows that rotavirus continues to contribute to the burden of diarrhoea in older children and adults, and thus there is a need for a better understanding of how all age groups contribute to the burden and transmission of rotavirus both pre- and post-vaccination.

The models provide satisfactory predictions of the trend towards increasingly winter seasonal peaks of rotavirus observed in both Dhaka and Matlab during the validation period (2013–2019). The pronounced shift towards annual epidemics compared to biannual epidemics observed during the model-fitting period is likely due to further declines in the birth rate. The crude birth rate decreased by about 2.5% between 2012 and 2019. Overall, the ability of the model to reliably predict rotavirus dynamics in Dhaka (urban) and Matlab (rural) provides confidence that the model can be used to investigate the impact of changes in birth rates on rotavirus patterns in other settings.

To identify effective vaccination strategies and evaluate vaccine impact, a better understanding of pre-vaccination rotavirus incidence is required. However, this is difficult if the patterns of rotavirus incidence are changing over time, as in the case of Dhaka. Our model provides a way of simulating rotavirus incidence in the absence of vaccination that accounts for the changing patterns in rotavirus epidemiology, thus providing a baseline against which to evaluate future vaccine impact. In addition, the model can also be used to evaluate the potential impact of different vaccination strategies to identify the optimal strategy for Bangladesh.

Our results add to the evidence of the importance of the changes in birth rates in controlling the temporal shift from biannual to annual rotavirus activity. The satisfactory agreement between the model and observed data from both Dhaka and Matlab achieved during model validation demonstrates the potential of the models to predict the future dynamics of rotavirus in the absence of vaccination. The lack of substantial decline in rotavirus cases in Dhaka and Matlab despite a consistent long-term decline in birth rate justifies the need for introduction of rotavirus vaccine in Bangladesh. The models we have developed and validated demonstrate the potential for use in evaluation of the impact of rotavirus vaccination in Bangladesh against the changing patterns of rotavirus incidence.

## Data Availability

The Institutional Review Board of icddr,b has the right to share data upon request. Data requests may be sent to Ms Armana Ahmed (aahmed@icddrb.org), head, Research Administration. Electronic supplementary material is available online [56].
